# Supplementary material for: Heparin-based hydrogel scaffolding alters the transcriptomic profile and increases the chemoresistance of MDA-MB-231 triple-negative breast cancer cells
Source: Biomater Sci. 2020 Feb 13;8(10):2786–96. doi: 10.1039/c9bm01481k (PMC7497406; doi:10.1039/c9bm01481k)
Supplement: Supplementary file 2 [file BM-008-C9BM01481K-s002.zip › Supplementary File 4/HP-BhydrogelvControl/Pathways/my_analysis.Gsea.1545201079907/HALLMARK_P53_PATHWAY.html]

Details for gene set HALLMARK\_P53\_PATHWAY[GSEA]

|  || Dataset | expr.class.cls#HYD\_versus\_CONTROL.class.cls#HYD\_versus\_CONTROL\_repos |
| Phenotype | class.cls#HYD\_versus\_CONTROL\_repos |
| Upregulated in class | HYD |
| GeneSet | HALLMARK\_P53\_PATHWAY |
| Enrichment Score (ES) | 0.19754215 |
| Normalized Enrichment Score (NES) | 0.8099284 |
| Nominal p-value | 0.88780487 |
| FDR q-value | 0.9735729 |
| FWER p-Value | 1.0 |
Table: GSEA Results Summary

  

Fig 1: Enrichment plot: HALLMARK\_P53\_PATHWAY      
 Profile of the Running ES Score & Positions of GeneSet Members on the Rank Ordered List

  

| PROBE | DESCRIPTION (from dataset) | GENE SYMBOL | GENE\_TITLE | RANK IN GENE LIST | RANK METRIC SCORE | RUNNING ES | CORE ENRICHMENT || 1 | PLK3 | na |  |  | 145 | 3.111 | 0.0150 | Yes |
| 2 | RAD9A | na |  |  | 300 | 2.671 | 0.0263 | Yes |
| 3 | PVT1 | na |  |  | 401 | 2.470 | 0.0390 | Yes |
| 4 | PDGFA | na |  |  | 484 | 2.361 | 0.0519 | Yes |
| 5 | IER5 | na |  |  | 719 | 2.098 | 0.0549 | Yes |
| 6 | FBXW7 | na |  |  | 855 | 1.991 | 0.0622 | Yes |
| 7 | NOTCH1 | na |  |  | 1056 | 1.835 | 0.0651 | Yes |
| 8 | PLXNB2 | na |  |  | 1060 | 1.833 | 0.0782 | Yes |
| 9 | IRAK1 | na |  |  | 1092 | 1.808 | 0.0898 | Yes |
| 10 | UPP1 | na |  |  | 1307 | 1.685 | 0.0908 | Yes |
| 11 | JAG2 | na |  |  | 1375 | 1.640 | 0.0992 | Yes |
| 12 | PPP1R15A | na |  |  | 1561 | 1.546 | 0.1007 | Yes |
| 13 | RAB40C | na |  |  | 1596 | 1.533 | 0.1101 | Yes |
| 14 | HEXIM1 | na |  |  | 1651 | 1.509 | 0.1182 | Yes |
| 15 | BAIAP2 | na |  |  | 1657 | 1.508 | 0.1289 | Yes |
| 16 | ADA | na |  |  | 1938 | 1.392 | 0.1243 | Yes |
| 17 | JUN | na |  |  | 1992 | 1.366 | 0.1314 | Yes |
| 18 | DDIT3 | na |  |  | 2346 | 1.225 | 0.1218 | Yes |
| 19 | POM121 | na |  |  | 2397 | 1.209 | 0.1280 | Yes |
| 20 | GADD45A | na |  |  | 2424 | 1.200 | 0.1353 | Yes |
| 21 | DEF6 | na |  |  | 2426 | 1.200 | 0.1440 | Yes |
| 22 | EPHA2 | na |  |  | 2560 | 1.156 | 0.1454 | Yes |
| 23 | ATF3 | na |  |  | 2583 | 1.150 | 0.1526 | Yes |
| 24 | XPC | na |  |  | 2677 | 1.124 | 0.1559 | Yes |
| 25 | TNFSF9 | na |  |  | 2848 | 1.072 | 0.1548 | Yes |
| 26 | SESN1 | na |  |  | 2905 | 1.062 | 0.1595 | Yes |
| 27 | TCHH | na |  |  | 3197 | 0.988 | 0.1514 | Yes |
| 28 | RXRA | na |  |  | 3265 | 0.972 | 0.1550 | Yes |
| 29 | WRAP73 | na |  |  | 3308 | 0.964 | 0.1598 | Yes |
| 30 | GPX2 | na |  |  | 3319 | 0.961 | 0.1662 | Yes |
| 31 | ST14 | na |  |  | 3433 | 0.931 | 0.1671 | Yes |
| 32 | SPHK1 | na |  |  | 3452 | 0.927 | 0.1729 | Yes |
| 33 | FDXR | na |  |  | 3632 | 0.886 | 0.1699 | Yes |
| 34 | DDB2 | na |  |  | 3676 | 0.875 | 0.1740 | Yes |
| 35 | FOXO3 | na |  |  | 3688 | 0.873 | 0.1798 | Yes |
| 36 | SLC3A2 | na |  |  | 3717 | 0.868 | 0.1846 | Yes |
| 37 | EPS8L2 | na |  |  | 3732 | 0.862 | 0.1901 | Yes |
| 38 | HRAS | na |  |  | 3805 | 0.850 | 0.1925 | Yes |
| 39 | CSRNP2 | na |  |  | 3875 | 0.838 | 0.1950 | Yes |
| 40 | OSGIN1 | na |  |  | 4056 | 0.800 | 0.1914 | Yes |
| 41 | NUPR1 | na |  |  | 4351 | 0.741 | 0.1813 | Yes |
| 42 | PLK2 | na |  |  | 4474 | 0.721 | 0.1801 | Yes |
| 43 | ABCC5 | na |  |  | 4570 | 0.697 | 0.1802 | Yes |
| 44 | AEN | na |  |  | 4661 | 0.683 | 0.1804 | Yes |
| 45 | KRT17 | na |  |  | 4866 | 0.647 | 0.1744 | Yes |
| 46 | BMP2 | na |  |  | 4891 | 0.643 | 0.1778 | Yes |
| 47 | VDR | na |  |  | 4898 | 0.641 | 0.1822 | Yes |
| 48 | RRP8 | na |  |  | 4903 | 0.640 | 0.1866 | Yes |
| 49 | BAX | na |  |  | 4924 | 0.638 | 0.1902 | Yes |
| 50 | AK1 | na |  |  | 4927 | 0.638 | 0.1948 | Yes |
| 51 | TSPYL2 | na |  |  | 4991 | 0.628 | 0.1960 | Yes |
| 52 | LIF | na |  |  | 5094 | 0.614 | 0.1951 | Yes |
| 53 | IFI30 | na |  |  | 5258 | 0.592 | 0.1908 | Yes |
| 54 | PMM1 | na |  |  | 5293 | 0.586 | 0.1933 | Yes |
| 55 | TRAF4 | na |  |  | 5328 | 0.579 | 0.1957 | Yes |
| 56 | RNF19B | na |  |  | 5374 | 0.573 | 0.1975 | Yes |
| 57 | RGS16 | na |  |  | 5502 | 0.552 | 0.1949 | No |
| 58 | HBEGF | na |  |  | 5567 | 0.542 | 0.1955 | No |
| 59 | ANKRA2 | na |  |  | 5724 | 0.522 | 0.1910 | No |
| 60 | CYFIP2 | na |  |  | 5787 | 0.515 | 0.1915 | No |
| 61 | RHBDF2 | na |  |  | 6007 | 0.484 | 0.1835 | No |
| 62 | TPD52L1 | na |  |  | 6047 | 0.480 | 0.1850 | No |
| 63 | FOS | na |  |  | 6167 | 0.461 | 0.1821 | No |
| 64 | INHBB | na |  |  | 6568 | 0.407 | 0.1640 | No |
| 65 | DCXR | na |  |  | 6794 | 0.384 | 0.1550 | No |
| 66 | RALGDS | na |  |  | 6974 | 0.364 | 0.1482 | No |
| 67 | MXD4 | na |  |  | 7123 | 0.345 | 0.1429 | No |
| 68 | KLF4 | na |  |  | 7185 | 0.341 | 0.1422 | No |
| 69 | KIF13B | na |  |  | 7273 | 0.329 | 0.1400 | No |
| 70 | RRAD | na |  |  | 7481 | 0.310 | 0.1314 | No |
| 71 | CDK5R1 | na |  |  | 7644 | 0.286 | 0.1250 | No |
| 72 | BTG2 | na |  |  | 7702 | 0.284 | 0.1240 | No |
| 73 | POLH | na |  |  | 7767 | 0.278 | 0.1227 | No |
| 74 | MKNK2 | na |  |  | 8109 | 0.246 | 0.1065 | No |
| 75 | PHLDA3 | na |  |  | 8190 | 0.234 | 0.1040 | No |
| 76 | IP6K2 | na |  |  | 8736 | 0.176 | 0.0767 | No |
| 77 | BAK1 | na |  |  | 9101 | 0.141 | 0.0586 | No |
| 78 | RAD51C | na |  |  | 9123 | 0.137 | 0.0584 | No |
| 79 | PRKAB1 | na |  |  | 9170 | 0.131 | 0.0570 | No |
| 80 | SOCS1 | na |  |  | 9324 | 0.113 | 0.0498 | No |
| 81 | TP63 | na |  |  | 9358 | 0.110 | 0.0488 | No |
| 82 | TRIB3 | na |  |  | 9434 | 0.099 | 0.0456 | No |
| 83 | NUDT15 | na |  |  | 9605 | 0.089 | 0.0373 | No |
| 84 | CCND2 | na |  |  | 9609 | 0.088 | 0.0378 | No |
| 85 | IL1A | na |  |  | 9651 | 0.080 | 0.0362 | No |
| 86 | ABAT | na |  |  | 9813 | 0.062 | 0.0282 | No |
| 87 | FUCA1 | na |  |  | 9835 | 0.060 | 0.0275 | No |
| 88 | PTPRE | na |  |  | 9844 | 0.059 | 0.0275 | No |
| 89 | ERCC5 | na |  |  | 9859 | 0.057 | 0.0272 | No |
| 90 | TGFB1 | na |  |  | 9937 | 0.048 | 0.0235 | No |
| 91 | PTPN14 | na |  |  | 9944 | 0.046 | 0.0235 | No |
| 92 | DGKA | na |  |  | 9953 | 0.045 | 0.0234 | No |
| 93 | CTSF | na |  |  | 9970 | 0.043 | 0.0229 | No |
| 94 | EPHX1 | na |  |  | 10016 | 0.038 | 0.0208 | No |
| 95 | ITGB4 | na |  |  | 10164 | 0.020 | 0.0132 | No |
| 96 | CASP1 | na |  |  | 10302 | 0.015 | 0.0062 | No |
| 97 | BLCAP | na |  |  | 10336 | 0.014 | 0.0045 | No |
| 98 | RETSAT | na |  |  | 10420 | 0.005 | 0.0002 | No |
| 99 | HDAC3 | na |  |  | 10467 | -0.001 | -0.0022 | No |
| 100 | PRMT2 | na |  |  | 10538 | -0.008 | -0.0058 | No |
| 101 | NINJ1 | na |  |  | 10608 | -0.017 | -0.0093 | No |
| 102 | TAP1 | na |  |  | 10646 | -0.022 | -0.0111 | No |
| 103 | SDC1 | na |  |  | 11066 | -0.073 | -0.0326 | No |
| 104 | CCND3 | na |  |  | 11490 | -0.109 | -0.0541 | No |
| 105 | CD81 | na |  |  | 11515 | -0.114 | -0.0545 | No |
| 106 | STEAP3 | na |  |  | 11653 | -0.132 | -0.0607 | No |
| 107 | CD82 | na |  |  | 11683 | -0.136 | -0.0613 | No |
| 108 | H2AFJ | na |  |  | 11690 | -0.137 | -0.0606 | No |
| 109 | CDKN1A | na |  |  | 11746 | -0.145 | -0.0624 | No |
| 110 | CCNK | na |  |  | 11824 | -0.154 | -0.0654 | No |
| 111 | SFN | na |  |  | 12185 | -0.198 | -0.0829 | No |
| 112 | TAX1BP3 | na |  |  | 12259 | -0.208 | -0.0852 | No |
| 113 | CTSD | na |  |  | 12285 | -0.211 | -0.0850 | No |
| 114 | VWA5A | na |  |  | 12806 | -0.258 | -0.1104 | No |
| 115 | FAS | na |  |  | 12828 | -0.261 | -0.1096 | No |
| 116 | NOL8 | na |  |  | 12894 | -0.271 | -0.1111 | No |
| 117 | SLC7A11 | na |  |  | 12949 | -0.278 | -0.1119 | No |
| 118 | ACVR1B | na |  |  | 13007 | -0.284 | -0.1128 | No |
| 119 | SERTAD3 | na |  |  | 13111 | -0.300 | -0.1161 | No |
| 120 | CGRRF1 | na |  |  | 13194 | -0.311 | -0.1181 | No |
| 121 | DRAM1 | na |  |  | 13242 | -0.315 | -0.1183 | No |
| 122 | LDHB | na |  |  | 13307 | -0.326 | -0.1193 | No |
| 123 | MXD1 | na |  |  | 13509 | -0.354 | -0.1273 | No |
| 124 | SEC61A1 | na |  |  | 13535 | -0.358 | -0.1260 | No |
| 125 | SAT1 | na |  |  | 13558 | -0.362 | -0.1245 | No |
| 126 | ZFP36L1 | na |  |  | 13646 | -0.378 | -0.1263 | No |
| 127 | APP | na |  |  | 13765 | -0.397 | -0.1296 | No |
| 128 | CCP110 | na |  |  | 14173 | -0.437 | -0.1479 | No |
| 129 | SLC19A2 | na |  |  | 14203 | -0.441 | -0.1462 | No |
| 130 | SP1 | na |  |  | 14317 | -0.467 | -0.1487 | No |
| 131 | TSC22D1 | na |  |  | 14324 | -0.468 | -0.1456 | No |
| 132 | ZMAT3 | na |  |  | 14337 | -0.469 | -0.1429 | No |
| 133 | SLC35D1 | na |  |  | 14339 | -0.469 | -0.1395 | No |
| 134 | MAPKAPK3 | na |  |  | 14391 | -0.475 | -0.1387 | No |
| 135 | WWP1 | na |  |  | 14407 | -0.478 | -0.1360 | No |
| 136 | DDIT4 | na |  |  | 14408 | -0.478 | -0.1326 | No |
| 137 | HSPA4L | na |  |  | 14452 | -0.483 | -0.1313 | No |
| 138 | TRAFD1 | na |  |  | 14581 | -0.503 | -0.1344 | No |
| 139 | MDM2 | na |  |  | 14595 | -0.505 | -0.1314 | No |
| 140 | PPM1D | na |  |  | 14788 | -0.536 | -0.1376 | No |
| 141 | PROCR | na |  |  | 14847 | -0.543 | -0.1367 | No |
| 142 | TRIAP1 | na |  |  | 15000 | -0.562 | -0.1406 | No |
| 143 | PITPNC1 | na |  |  | 15035 | -0.567 | -0.1383 | No |
| 144 | APAF1 | na |  |  | 15381 | -0.631 | -0.1518 | No |
| 145 | TCN2 | na |  |  | 15404 | -0.635 | -0.1483 | No |
| 146 | HMOX1 | na |  |  | 15431 | -0.638 | -0.1451 | No |
| 147 | ABHD4 | na |  |  | 15481 | -0.647 | -0.1429 | No |
| 148 | RAP2B | na |  |  | 15504 | -0.651 | -0.1394 | No |
| 149 | CDKN2AIP | na |  |  | 15782 | -0.699 | -0.1488 | No |
| 150 | BTG1 | na |  |  | 15833 | -0.711 | -0.1463 | No |
| 151 | RPL18 | na |  |  | 15882 | -0.720 | -0.1436 | No |
| 152 | PCNA | na |  |  | 15928 | -0.728 | -0.1407 | No |
| 153 | STOM | na |  |  | 15937 | -0.731 | -0.1358 | No |
| 154 | EI24 | na |  |  | 16083 | -0.766 | -0.1378 | No |
| 155 | DNTTIP2 | na |  |  | 16169 | -0.782 | -0.1366 | No |
| 156 | ISCU | na |  |  | 16260 | -0.800 | -0.1355 | No |
| 157 | RB1 | na |  |  | 16313 | -0.813 | -0.1323 | No |
| 158 | HINT1 | na |  |  | 16615 | -0.878 | -0.1418 | No |
| 159 | TP53 | na |  |  | 16860 | -0.944 | -0.1477 | No |
| 160 | GM2A | na |  |  | 17215 | -1.057 | -0.1587 | No |
| 161 | HIST1H1C | na |  |  | 17481 | -1.146 | -0.1643 | No |
| 162 | TXNIP | na |  |  | 17600 | -1.197 | -0.1618 | No |
| 163 | RPL36 | na |  |  | 17619 | -1.206 | -0.1539 | No |
| 164 | NDRG1 | na |  |  | 17623 | -1.208 | -0.1453 | No |
| 165 | RPS27L | na |  |  | 17831 | -1.300 | -0.1467 | No |
| 166 | TOB1 | na |  |  | 17952 | -1.348 | -0.1432 | No |
| 167 | TM7SF3 | na |  |  | 18054 | -1.419 | -0.1382 | No |
| 168 | RCHY1 | na |  |  | 18153 | -1.468 | -0.1327 | No |
| 169 | F2R | na |  |  | 18280 | -1.550 | -0.1280 | No |
| 170 | TGFA | na |  |  | 18621 | -1.808 | -0.1328 | No |
| 171 | FAM162A | na |  |  | 18694 | -1.889 | -0.1228 | No |
| 172 | PERP | na |  |  | 18900 | -2.167 | -0.1178 | No |
| 173 | TPRKB | na |  |  | 18945 | -2.253 | -0.1038 | No |
| 174 | RPS12 | na |  |  | 18989 | -2.312 | -0.0892 | No |
| 175 | CCNG1 | na |  |  | 19060 | -2.553 | -0.0743 | No |
| 176 | S100A10 | na |  |  | 19095 | -2.708 | -0.0564 | No |
| 177 | S100A4 | na |  |  | 19100 | -2.731 | -0.0368 | No |
| 178 | TM4SF1 | na |  |  | 19118 | -2.862 | -0.0168 | No |
| 179 | VAMP8 | na |  |  | 19121 | -2.887 | 0.0040 | No |
Table: GSEA details [plain text format]

  

Fig 2: HALLMARK\_P53\_PATHWAY      
 Blue-Pink O' Gram in the Space of the Analyzed GeneSet

  

Fig 3: HALLMARK\_P53\_PATHWAY: Random ES distribution      
 Gene set null distribution of ES for **HALLMARK\_P53\_PATHWAY**
